# Supplementary material for: An exploration of the costs of family and group conferencing pathways in adult social care and mental health: A scenario-based cost analysis
Source: PLoS One. 2025 Dec 3;20(12):e0326829. doi: 10.1371/journal.pone.0326829 (PMC12674543; doi:10.1371/journal.pone.0326829)
Supplement: S1 Table — (DOCX) [file pone.0326829.s001.docx]

**S1 Table.** **Sensitivity results: Probability of advancing to the full FGC pathway (LA/NHS perspective)**

| **Stage of FGC / probability of advancing to the full FGC** | **Adult Social Care** | | | **Mental Health Care** | | |
| --- | --- | --- | --- | --- | --- | --- |
|  | **65%** | **75%** | **85%** | **65%** | **75%** | **85%** |
| Number of network members | 3 | 3 | 3 | 3 | 3 | 3 |
| Number of professional staff | 2 | 2 | 2 | 2 | 2 | 2 |
| Duration of Conference | 4 | 4 | 4 | 4 | 4 | 4 |
| **Total (£) 2022-23 prices** | 1160 | 1246 | 1333 | 1313 | 1406 | 1499 |
| **Adjusted to March 2025 prices** | 1194 | 1283 | 1372 | 1352 | 1447 | 1543 |

Notes: If 65% of FGC referrals follow the full FGC pathway (Scenario A) and 35% follow the non-conference pathway (Scenario E), the expected cost per case accepted into the FGC service would be: £1462*0.65 + £599*0.35 = £979.10.
