# Supplementary material for: An exploration of the costs of family and group conferencing pathways in adult social care and mental health: A scenario-based cost analysis
Source: PLoS One. 2025 Dec 3;20(12):e0326829. doi: 10.1371/journal.pone.0326829 (PMC12674543; doi:10.1371/journal.pone.0326829)
Supplement: S2 Tables — (DOCX) [file pone.0326829.s002.docx]

S2 Tables: Costing for the Referral Stage

**S2A Table: Costs of FGC pathways (including Referral stage) in five scenarios (LA/NHS perspective)**

| **Stage of FGC** | **Scenarios** | | | | |
| --- | --- | --- | --- | --- | --- |
|  | A | B | C | D | E |
|  | / | inc. advocate | inc. interpreter | exc. food | Non-conference |
|  | **Panel A: Adult Social Care** | | | | |
| **1.Referral to FGC** | 106 | 106 | 106 | 106 | 106 |
| **2.Preparation** | 599 | 683 | 659 | 599 | 599 |
| **3.Conference** | 757 | 900 | 888 | 708 | 0 |
| **4.Review meetings** | 106 | 190 | 166 | 106 | 0 |
| **Total (£) 2022-23 prices** | 1568 | 1879 | 1819 | 1519 | 705 |
| **Relative to baseline** | 100% | 120% | 116% | 97% | 45% |
| **Adjusted to March 2025 prices** | 1614 | 1934 | 1873 | 1564 | 726 |
|  | **Panel B: Mental Health care** | | | | |
| **1.Referral to FGC** | 128 | 128 | 128 | 128 | 128 |
| **2.Preparation** | 709 | 793 | 769 | 709 | 709 |
| **3.Conference** | 801 | 980 | 932 | 752 | 0 |
| **4.Review meetings** | 128 | 212 | 188 | 128 | 0 |
| **Total (£) 2022-23 prices** | 1766 | 2113 | 2017 | 1717 | 837 |
| **Relative to baseline** | 100% | 120% | 114% | 97% | 47% |
| **Adjusted to March 2025 prices** | 1818 | 2175 | 2076 | 1768 | 862 |

Notes: The differences between adult social care and mental health settings are due to the assumed role of the referrer (a social worker in adult social care and a care coordinator in mental health) and the unit cost of the FGC coordinator.

**S2B: Costs of FGC pathways in five scenarios in mental health care with a psychiatric consultant making referral (NHS perspective)**

| **Stage of FGC** | **Scenarios in Mental Health Care** | | | | |
| --- | --- | --- | --- | --- | --- |
|  | A | B | C | D | E |
|  | (baseline) | inc. advocate | inc. interpreter | exc. food | Non-conference |
|  | **Referrer as a psychiatric consultant** | | | | |
| **1.Referral to FGC** | 207 | 207 | 207 | 207 | 207 |
| **2.Preparation phase** | 709 | 793 | 769 | 709 | 709 |
| **3.Conference phase** | 801 | 980 | 932 | 752 | 0 |
| **4.Review meetings** | 128 | 212 | 188 | 128 | 0 |
| **Total (£) 2022-23 prices** | 1845 | 2192 | 2096 | 1796 | 916 |
| **Relative to baseline** | 100% | 119% | 114% | 97% | 50% |
| **Adjusted to March 2025 prices** | 1899 | 2257 | 2158 | 1849 | 943 |

Notes: In mental health setting, we assumed the referrer is a psychiatric consultant. All the other assumptions are the same as in Table 7 (main paper).
